# A Liquid Metal Temperature Detection System Based on Multi-Node Sapphire Fiber Sensor

**DOI:** 10.3390/s23094318

**Published:** 2023-04-27

**Authors:** Gao Wang, Chengyuan Qian, Peng Sun, Zhiling Li, Guofeng Chen, Bingyin Wang, Hanchang Zhou, Junzhi Yu

**Affiliations:** 1 State Key Laboratory of Dynamic Measurement Technology, North University of China, Taiyuan 030051, China; wanggao@nuc.edu.cn (G.W.);; 2 Inner Mongolia Power Machinery Institute, Hohhot 010010, China; 3 School of Information and Communication Engineering, North University of China, Taiyuan 030051, China; 4 Department of Advanced Manufacturing and Robotics, College of Engineering, Peking University, Beijing 100871, China; 5 Science and Technology on Integrated Information System Laboratory, Institute of Software, Chinese Academy of Sciences, Beijing 100190, China

**Keywords:** ultrasonic temperature measurement, aluminum liquid, sapphire fiber

## Abstract

In order to accurately detect the temperature of molten aluminum and overcome the adverse influence of high temperature and corrosiveness on the sensing results, a temperature detection system based on a multi-node sapphire fiber sensor was proposed and developed. Through the structural parameter design of the fiber sensor, the scheme of utilizing the 0.7 mm diameter fiber and 0.5 mm groove was formulated. Simulation and analysis were carried out to determine the ultrasonic response distribution of the signal passing through the whole fiber sensor. The results indicate that the system is capable of distinguishing test signals from various positions and temperatures. Following the completion of the static calibration, the temperature of the molten aluminum was observed in real-time, and the data of the temperature measurements conducted at the two groove locations were compared. According to the obtained results, the test accuracy was greater than 1 degree Celsius and the temperature test stability was good, laying a solid foundation for the potential development of temperature measurement devices.

## 1. Introduction

Metal casting plays a role in 90% of all manufactured goods [1]. There are a large number of aluminum products in the manufacturing industry [2,3,4,5,6,7,8], and efficient production process control can reduce production costs and improve product quality. In the process of manufacturing casting products, the temperature control of molten aluminum is a key factor [3,8,9,10,11]. In this sense, it is of great significance to control the temperature of molten aluminum to improve the performance of castings.

The practical infrared temperature measurement method and the k-type armored thermocouple measurement method are currently the two most commonly used ways to detect the temperature of molten metal [9,12,13]. Heeley et al. [12] developed a new infrared thermometer based on an Indium Arsenide Antimony (InAsSb) photodiode, a trans-impedance amplifier, and a sapphire fiber optic cable. The prototype thermometer was capable of measuring temperatures between 200 °C and 1000 °C with a sapphire fiber optic cable coupling to high-temperature conditions. The convenient infrared detection method is a non-contact measurement, which has the advantages of no pollution and long life, but the measurement error is large, and the temperature value of the internal molten aluminum cannot be accurately obtained. Although the k-type armored thermocouple method is simple, it is easy to damage the metal tube due to the strong corrosion of the high-temperature aluminum liquid, resulting in inaccurate measurement results. Bramanti et al. [14] presented an acoustic pyrometry method based on measuring the times-of-flight of acoustic waves along a number of straight paths in a cross-section of the boiler to reconstruct temperature maps inside power plant boilers. Young et al. [15] computed the acoustic constants and temperature values of typical fuel combustion products. Their work demonstrated that acoustic pyrometry offered a powerful alternative with the potential to greatly increase measurement accuracy. Lu et al. [16] proposed an acoustic path refraction algorithm for computed tomography and used the least-squares method, which is capable of considering refraction effects to reconstruct temperature distribution. He et al. [17] built a test device based on acoustic measurements, and the test results were satisfactory. Tian [18] utilized the method of combining the wavelet frame theory and filter to process the signal, and the reconstructed results were basically consistent with the actual gas distribution in the furnace. The integrated ultrasonic transducers (IUTs) and flexible ultrasonic transducers (FUTs) developed by Kobayashi et al. [19] were used for non-destructive testing at high temperatures, and their operation frequencies ranged from 4.4 to 10.7 MHz with signal-to-noise ratios-above-20 dB. Bufetova et al. [20,21] experimentally obtained temperature distribution along a source rod and molten zone in sapphire and yttrium–aluminum garnet single-crystal fibers under a growing LHPG process for the first time. The results obtained could be used for the improvement of crystal fiber growth methods.

All the work mentioned above provides the foundation for an ultrasonic thermometer. Based on the principle of ultrasonic temperature measurement, we adopted sapphire fiber as a sensitive material to develop a multi-node fiber temperature sensor in this work. As Al_2_O_3_ makes up the fiber, there is no reaction with the liquid aluminum. It is capable of achieving long-time measurement goals. Using sapphire fibers and the ultrasonic temperature measurement technique, we created a multi-node temperature sensor that can accurately monitor the temperature of molten aluminum.

## 2. System Design

**Figure 1:**
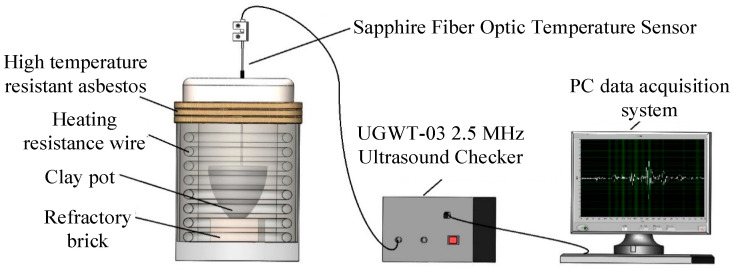
Schematic diagram of the temperature measurement system.

The molten aluminum temperature measurement system based on the principle of an ultrasonic temperature measurement was mainly composed of a 7500 W aluminum alloy resistance melting furnace (250 mm × 300 mm), a 10# graphite clay pot (137 mm × 100 mm × 180 mm), sapphire fiber temperature sensor (500 mm), UGWT-03 type 2.5 MHz ultrasonic detector, and a PC data acquisition system of 100 MHz. The overall temperature measurement system is illustrated in Figure 1.

In the test system, the ring-shaped resistance wire in the inner wall of the furnace generated heat after the heating power of the molten aluminum was generated by the control cabinet, and the aluminum blocks in the clay pot were melted by heat exchange. When the rated temperature of the control cabinet reached 640 °C, the aluminum blocks were essentially melted in the furnace. As the given temperature continued to increase, the temperature of the molten aluminum gradually increased after thermal equilibrium. The maximum given temperature value of the aluminum alloy resistance furnace used in the experiment was 740 °C, and the resistance furnace would be damaged due to excessive loading power if this value was exceeded.

## 3. Sensor Design and Preparation

### 3.1. Sensitive Area Parameters Design

**Figure 2:**
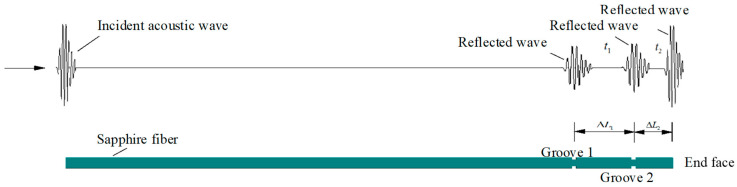
Schematic diagram of the principle of ultrasonic temperature measurement.

According to the principle of ultrasonic temperature measurements, two grooves were designed on the temperature measurement sensitive element, and the sound velocity at different temperatures was calculated by measuring the delay data t_1_ and t_2_ under a certain propagation distance: ΔL_1_ and ΔL_2_ between groove 1 and groove 2 and the end face as illustrated in Figure 2. The groove design caused the pulse wave to generate impedance along the propagation path, forming a reflected wave. In addition, the diameter of the groove directly affects the amplitude change in the reflected wave at the variable cross-section. Therefore, it is necessary to determine the size of sensitive area parameters through an accurate calculation. According to the theoretic results in [22], the relationship between the diameter of the propagation rod of sensitive materials and the reflection coefficient can be described as follows:

(1) $$\frac{d_{2}}{d_{1}}=\sqrt{\frac{1+R}{1-R}}$$

where *d*_1_ and *d*_2_ are the diameters on both sides of the propagation rod variable cross-section, and *R* is the acoustic reflection coefficient at the variable cross-section.

Note that *R* usually ranges from 0.2 to 0.3 [22], and *R* = 0.25 was selected in this work. The diameter of the groove in the sensitive area can be calculated according to the relationship between the diameter of the propagation rod and the reflection coefficient in Equation (1). The diameter of the sapphire fiber propagation rod was 0.7 mm, and the diameter of the groove was 0.5 mm after calculation using the formula mentioned above.

### 3.2. Sapphire Fiber Preparation

**Figure 3:**
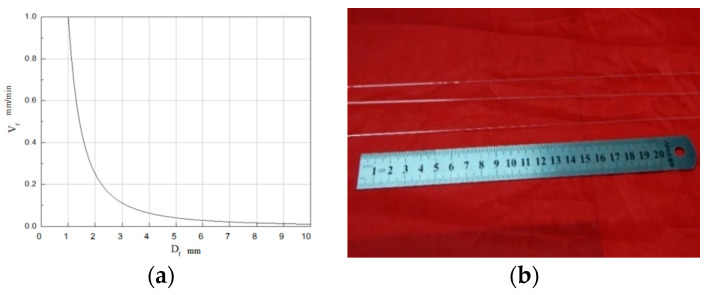
Sapphire fiber preparation. (**a**) Relationship between growth rate and fiber diameter; (**b**) Sapphire fiber drawn by LHPG.

Single-crystal sapphire fibers were grown by a laser-heated pedestal growth (LHPG) technique. When drawing a single-crystal fiber with a ring-focusing laser heating system, in order to ensure the uniformity and diameter of the straightened fiber, the stepper motor is usually controlled to ensure the uniform rise in speed *v_f_* of the seed crystal. At the same time, the rising speed vs. the source rod was altered to obtain the desired fiber diameter *D_f_*.

(2) $$D_{f}=\sqrt{\frac{v_{s}}{v_{f}}}D_{s}$$

where *D_s_* is the diameter of the source rod. For a single crystal fiber with a known diameter *D*, when the rising speed of the source rod is constant, the mass *m* of the crystal fiber growing per unit time can be obtained:

(3) $$m=\rho v\pi D^{2}/4$$

where *v* is the pulling speed of the seed crystal, and *ρ* is the density of the crystal. During the single-crystal fiber drawing process, the stability of the melting zone has a certain influence on the growth of the single-crystal fiber. The change in the melting zone directly affects the temperature gradient inside the crystal, forming a non-uniform temperature zone. The corresponding relationship curve between growth rate and fiber diameter is shown in Figure 3a. As can be observed, the larger the diameter of the fiber to be obtained, the smaller the growth rate value is when controlled by the setting.

In the actual drawing process, the size of the melting zone needs to be calculated accurately. In order to obtain a single-crystal fiber with good quality, the rising speed of the source rod and seed crystal needs to be adjusted. The diameters of the single-crystal fiber and source rod should satisfy the following formula:

(4) $$\frac{D_{f}}{D_{s}}=\frac{1}{2}-\frac{1}{3}.$$

Then, the length of the melting zone can become:

(5) $$L=K(D_{f}+D_{s}).$$

In general, coefficient *K* ranged from 0.6 to 0.8 for the grown single-crystal fiber. The tail of the single-crystal fiber grown through the melting zone was connected to the source rod. Therefore, the quality of the formed single-crystal fiber could be improved by the effective control of the melting zone. The sapphire single-crystal fiber drawn by LHPG in this paper is shown in Figure 3b.

## 4. Multi-Node Sensing Simulation Analysis

### 4.1. Model Construction and Meshing

**Figure 4:**
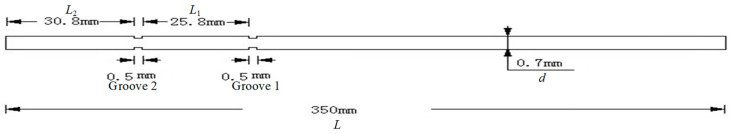
Structure parameters of sapphire fiber.

On the basis of the previous sensitive area parameters design, the structure of the sapphire fiber in Figure 4 was established. As illustrated in Figure 4, the total length L of the sapphire fiber was 350 mm, the diameter d was 0.7 mm, the length of the two grooves were both 0.5 mm, and the temperature-sensitive lengths *L*_1_ and *L*_2_ of the material were 25.8 mm and 30.8 mm, respectively.

**Figure 5:**
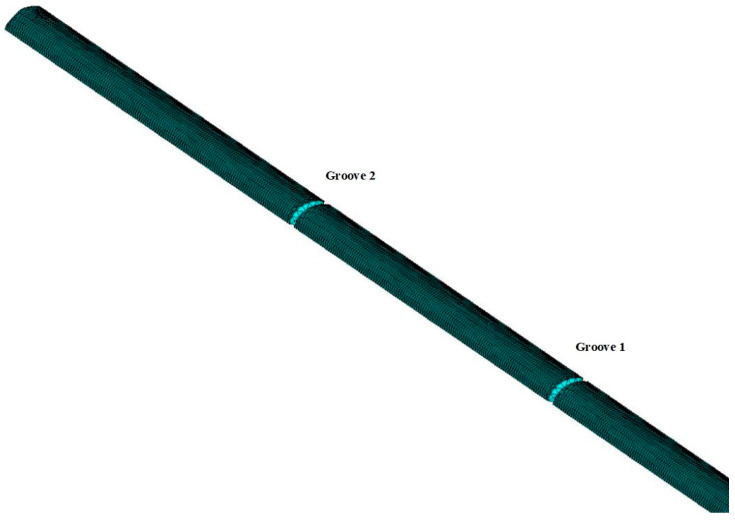
Meshing of fiber sensor.

In the process of the simulation study of ultrasonic propagation characteristics in the sapphire fiber waveguide, in order to ensure an error within 5% and obtain clear reflected signals, the grid division had a certain relationship with the excitation frequency. That is, the grid length of the divided cells was generally less than 1/8 of the excitation wavelength. Based on the above conditions, the central frequency f_0_ of the excitation terminal and the size L_E_ of the grid cell selected were 2.5 MHz and 0.2 mm, respectively. The meshed model diagram is illustrated in Figure 5.

### 4.2. Simulation and Analysis of Ultrasonic Transmission Characteristics

**Figure 6:**
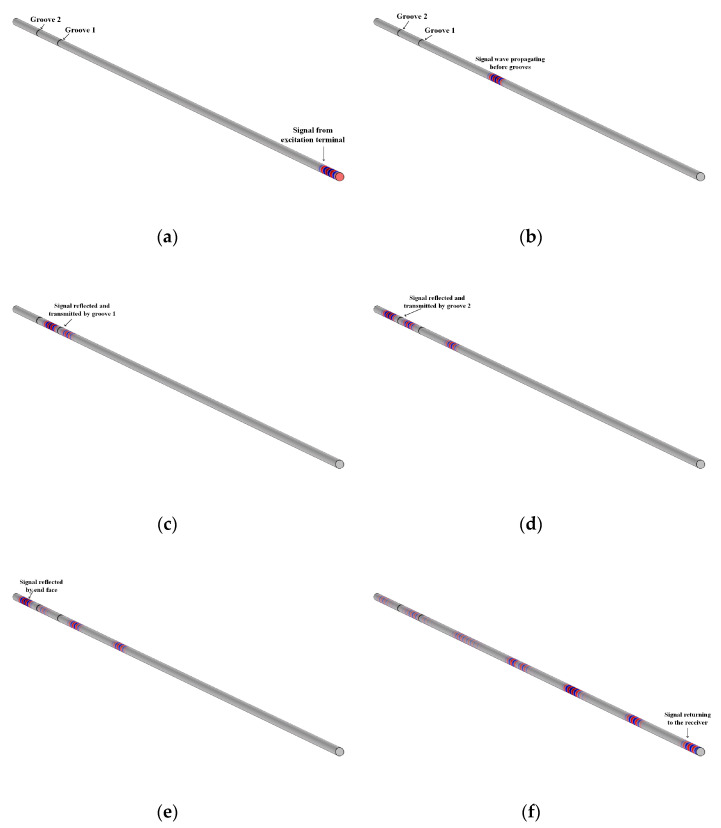
Meshing simulation of sapphire fiber sensor. (**a**) The excitation terminal generates a signal; (**b**) The signal wave propagates before grooves; (**c**) The signal in the first groove; (**d**) The signal in the second groove; (**e**) The signal in the top reflection; (**f**) The signal returned to the receiver.

The research object is a longitudinal propagation of the sound wave in sapphire fiber. Therefore, an axial direction constraint could be applied to the fiber excitation end, and the excitation wave load along the axis could be applied to the node group of the excitation end. After the simulation of the ultrasonic transmission characteristics in the sapphire fiber propagation rod, the results were generally observed in two ways. One was to visually observe the dynamic process of the excitation wave in the fiber in post-processing, and the other was to observe the amplitude of the waveform reflected back by groove 1 and groove 2 through the coordinate curve. The dynamic process of the excitation wave propagation obtained is shown in Figure 6.

It can be clearly seen from Figure 6 that ultrasonic waves were transmitted and reflected at the grooves in the sapphire fiber, and the reflected waves at the grooves and the end face were received by the ultrasonic transducer. The signal waveforms reflected from grooves 1 and 2 and the end face were collected, and the velocity value at the corresponding temperature could be obtained by calculating the delay data between the waveforms.

**Figure 7:**
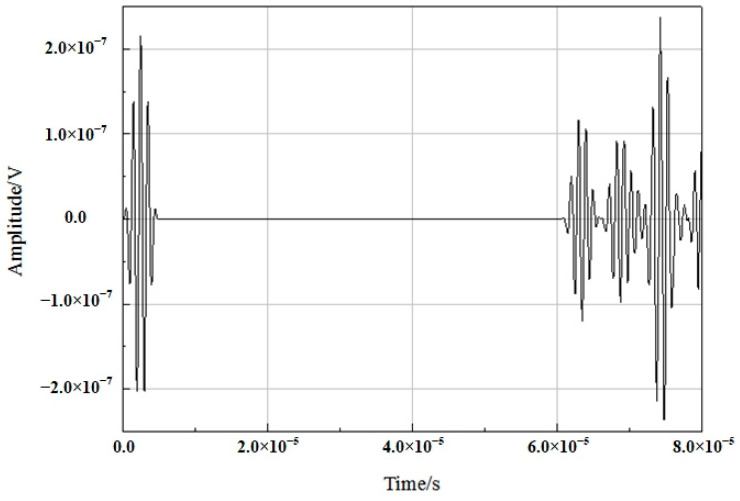
Waveform over time.

The node was selected from the receiving end of the waveguide rod, and the change in its own wave displacement with time could be observed in the way of a coordinate curve before the sound velocity was calculated according to the change in the displacement data of the propagation rod. The propagation characteristic curve is displayed in Figure 7.

It can be clearly seen from the figure above that the ultrasonic waveform changed with time in the sapphire fiber. After loading the excitation wave at the excitation end, the first wave packet is the waveform was reflected back by the wave transmitted to groove 1; the second wave packet is the waveform was reflected back by the wave transmitted to groove 2, and so on. It should be noted that if the segment lengths of grooves 1 and 2 are set to be equal, then the secondary waveform reflected back by propagation will be superimposed with the primary reflected wave, resulting in errors in analysis. Therefore, in order to avoid the occurrence of this phenomenon, when designing the material parameters, the size of the two grooves should be different, and the amplitude of the waveform after secondary reflection should not be the same, which is conducive to quantitative calculation and the analysis of delayed data.

## 5. Experiments

### 5.1. Static Calibration Experiment

**Figure 8:**
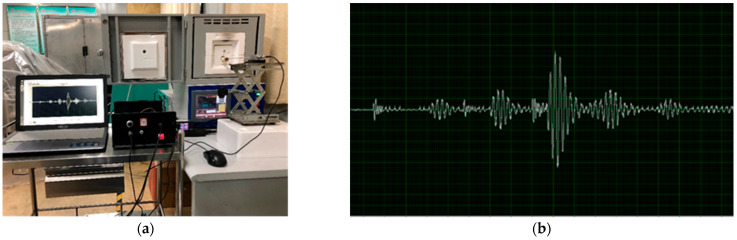
Calibration system and data acquisition results. (**a**) Laboratory calibration system; (**b**) Data acquisition results.

Concerning the temperature measuring system, before measuring the temperature of liquid aluminum, it was necessary to carry out a static calibration experiment on the designed sapphire fiber temperature sensor to check the amplitude of the sensor waveform, delay data, structural performance stability, and so on. During the laboratory calibration process, a 1600 °C high-temperature resistance furnace was used for data calibration. In the furnace, double rows of silicon-molybdenum rods were used for heating, and the surrounding areas were insulated with high-temperature firebricks to form a 100 mm × 100 mm × 100 mm temperature zone. Since the heating time process was set to hold for 5 min at integer temperature points, the internal temperature zone could be approximated as a constant temperature field. The sensor was placed in the constant temperature field together with a standard platinum-rhodium thermocouple. The corresponding data were collected and recorded every time the thermocouple changed by 100 °C. The calibration system and data acquisition results are shown in Figure 8.

**Figure 9:**
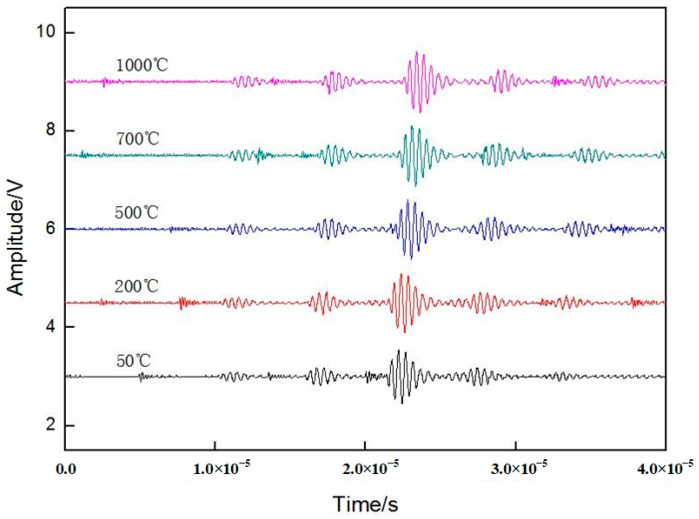
Delay data and waveforms at different temperatures.

The distance between groove 1 and groove 2 and the end face of the sapphire fiber *multi-node* temperature sensor was a known determinate value L_1_ and L_2_. The groove design caused the pulse wave to generate impedance along the propagation path, forming a reflected wave. The ultrasonic waves were transmitted and reflected at grooves in the sapphire fiber, and the reflected waves at the grooves and the end face were received by an ultrasonic transducer. By collecting the waveform reflected from groove 1 and groove 2 at different temperatures, the relationship between the delayed data and the current temperature value could be calculated. By calibrating the delay data, the delay data from room temperature to 1600 °C and the relationship curve between the temperature and delay data could be obtained. The waveforms at 50, 200, 500, 700, and 1000 °C were selected from the calibration data for fitting. As shown in Figure 9, the time delay between the grooves increased as the temperature increased, and the waveform gradually moved backward.

**Figure 10:**
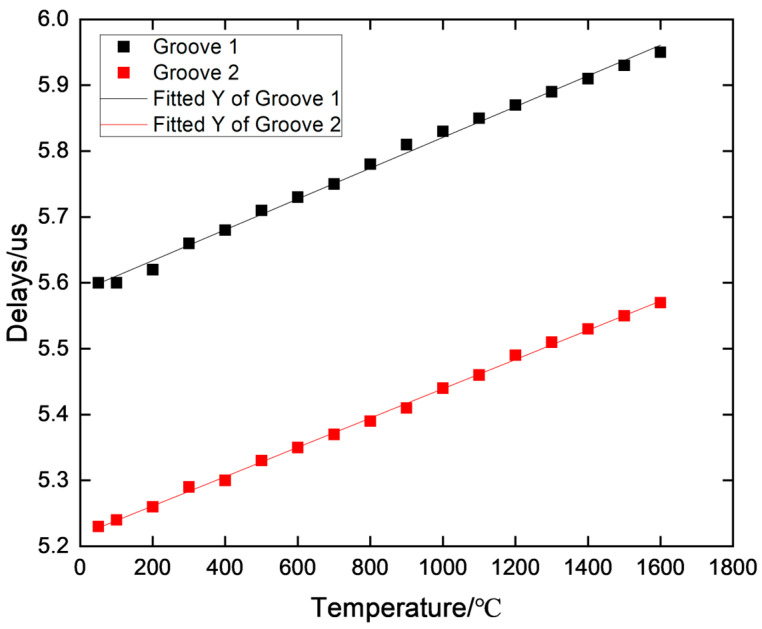
Delay data at different temperatures.

The calibration data were processed at different temperatures, and the results are shown in Figure 10. The linearity and error of groove 1 were 0.998 and 8.72 × 10^−4^, respectively. For groove 2, the linearity and error were 0.999 and 2.32 × 10^−4^, respectively. It can be noted through the calibration data from multiple tests there was little difference in the test results of the sensor, indicating that the sensor had good stability. Thus, the fabricated sensor could be used for temperature measurements.

### 5.2. Temperature Measurement Experiment of Molten Aluminum

In the actual temperature measurement process, the sapphire fiber temperature sensor needed to be inserted into the molten aluminum liquid for measurement, and the minimum temperature of aluminum in the molten state was about 640 °C. The maximum upper limit temperature of the aluminum alloy resistance furnace used in this experiment could reach 740 °C. Considering the above test conditions, two different temperature tests were carried out during the actual temperature measurement process.

- Process 1: The sensor was inserted into liquid aluminum in the lowest molten state to achieve thermal balance, and the data were continuously collected to obtain the time response curve of the sensor.
- Process 2: The temperature value was set to the upper threshold through the control cabinet, and continuous data collection was conducted during the heating process of the molten aluminum liquid. Since this process was heated by means of a furnace wall-air-dry pot heat exchange, the heating process was relatively slow, and long-term continuous data acquisition was required to obtain the heating curve of the temperature over time.

**Figure 11:**
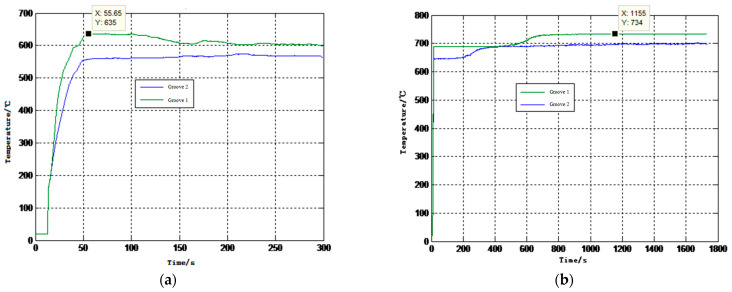
Temperature vs. time curve. (**a**) Temperature curve in the lowest molten state during process 1; (**b**) Temperature curve at the upper threshold during process 2.

In addition, multiple single-point temperature measurements of liquid aluminum at the highest temperature value were performed. The temperature of liquid aluminum was measured according to the above temperature measurement process, and the data results were processed and analyzed to obtain the temperature curve over time, as shown in Figure 11.

According to the change curve of the temperature in Figure 11a, it can be concluded that when the sensor was inserted into the molten aluminum, the temperature began to rise rapidly. With a further increase in time, the temperature of the sensor basically kept fluctuating horizontally. In addition, it can be seen from the temperature rise curve in this figure that the sapphire fiber multi-node temperature sensor reached a thermal equilibrium state after being inserted into the molten aluminum liquid for 55.65 s. Since the molten aluminum transfers heat through heat exchange, it can be noted from the experimental data in the above figure that there was a temperature difference between the two sensitive areas of groove 1 and groove 2 in the longitudinal direction of molten aluminum. Among them, the position of groove 1 was closer to the bottom of the dry pot, and the temperature measured after the heat balance was higher; groove 2 was close to the liquid surface of liquid aluminum, and the temperature was relatively low, indicating that the temperature distribution inside liquid aluminum was not uniform during the heat exchange heating process.

After the completion of the above process 1, the temperature of the molten aluminum liquid in the dry pot continued to rise over time by adjusting the rated temperature of the control cabinet, as shown in Figure 11b. After about 15 min, the temperature value reached 734 °C (the upper peak temperature of the control cabinet was 740 °C. If the temperature continued to rise, the control cabinet would be damaged due to excessive power). After the sapphire fiber temperature sensor was measured in molten aluminum for a long time, the wave amplitude did not change, and no corrosion was found on the fiber surface in the temperature-sensitive area. It can be seen that the sapphire fiber multi-node temperature sensor based on the principle of ultrasonic temperature measurement can be used as a new method to measure the temperature of molten aluminum.

**Figure 12:**
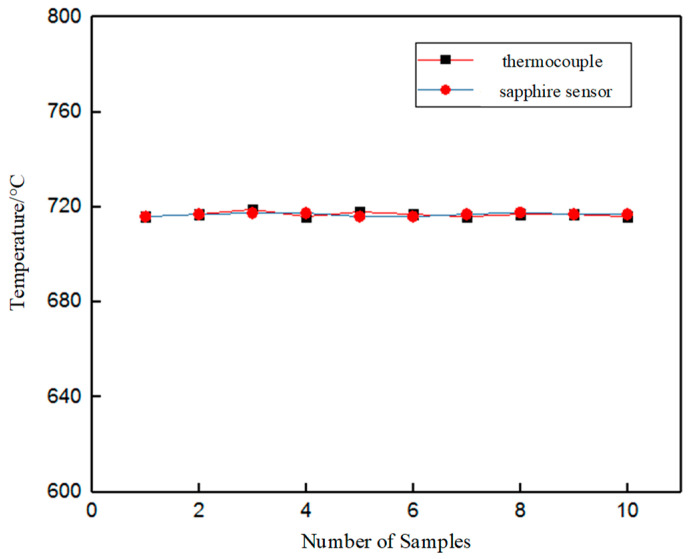
Comparison of the sapphire fiber sensor and the K-type thermocouple.

In the temperature measurement experiment of molten aluminum, apart from the above continuous temperature measurement relationship over time, multiple single-point temperature tests were carried out when the control cabinet was set at 740 °C. At the same time, the armored K-type thermocouple was inserted into the aluminum liquid for testing and comparison, and the mean value comparison curve between the thermocouple and the sensor was obtained, as shown in Figure 12.

By comparing the average temperature of the two sensors, as shown in the figure, it can be concluded that the sapphire fiber multi-node temperature sensor effectively carried out distributed temperature acquisition at different positions inside the molten aluminum liquid. According to the results, the test accuracy was greater than 1 degree Celsius and the temperature test stability was good.

### 5.3. Discussions

During the process of delayed data acquisition, it can be seen that there were some interference waveforms at the system acquisition interface. According to the data acquisition results of Figure 8 and Figure 9, the external interference noise had little effect on the extraction of the delayed information. However, when the sensor measured the temperature in an electromagnetic field environment, the generated interference wave overlaps with the waveform reflected back from the groove, which causes trouble in data analysis and leads to calculation errors. Therefore, in the follow-up study, it will be necessary to filter the acquired waveform to reduce errors in the calculation process.

## 6. Conclusions

In this paper, a sapphire fiber multi-node temperature sensor was investigated and manufactured based on the principle of ultrasonic temperature measurement. It can coexist well with molten aluminum without reacting. Based on the parameter design and simulation analysis, the static calibration test of the sensor from normal temperature to 1600 °C was completed, and the sound wave transmission curves at different temperatures were obtained. The curves of ultrasonic waves in the sapphire fiber propagating rod were obtained, and the delay time between the waves reflected from the groove and the end face at different temperatures was deduced. In the molten metal temperature test experiment of molten metal, the data results of the continuous acquisition of the sapphire fiber multi-node temperature sensor in molten aluminum were analyzed. The feasibility of the system was verified for the measurement of metal liquid temperature. The test accuracy can still be further improved. In follow-up work, we plan to optimize the filtering method of the acquired waveform to reduce the calculation error.

## Data Availability

Not applicable.
